# Repertory to Hering’s Condensed Materia Medica

**Published:** 1889-09

**Authors:** 


### Repertory to Hering's Condensed Materia Medica. Published by the Homœopathic Medical Society of Pennsylvania.

The purpose of this work is to give a repertorial index to Hering's volume, a much-used book. The Society hopes to be able to make the index complete; it is arranged by sections. The present issue includes such sections as the Lower Extremities, by Dr. J. L. Ferson; Male Sexual Organs, by Dr. C. Weaver; Appetite, Thirst, Desires, and Aversions, by Dr. Edward Cranch; Outer Chest, by Dr. S. F. Shannon; Stomach, by Dr. A. P. Bowie; the Aggravation of Mental Symptoms, by Dr. Z. T. Miller; Tongue, by Dr. E. Fornias; Symptoms Occurring during Pregnancy, by Dr. T. J. Gramm; Heart, by Dr. E. R. Snader.

The value of this work is greatly decreased by the poor arrangement, which makes the finding of any symptom very difficult.

Single copies may be purchased of the Secretary, Dr. Edward R. Snader, 140 North Twentieth Street, Philadelphia. Price, $2.00.

